# A case of mixed adenoneuroendocrine carcinoma of the pancreas: Immunohistochemical analysis for histogenesis

**DOI:** 10.1097/MD.0000000000006225

**Published:** 2017-03-03

**Authors:** Masaru Murata, Hidekazu Takahashi, Moyuru Yamada, Misa Song, Masahiro Hiratsuka

**Affiliations:** a Department of Surgery; b Department of Pathology, Itami City Hospital, Itami-shi, Hyogo, Japan.

**Keywords:** chromogranin A, histogenesis, mixed adenoneuroendocrine carcinoma, neuron-specific enolase, p53, synaptophysin

## Abstract

**Rationale:**

Tumors with multiple histological features, such as adenocarcinomas and neuroendocrine carcinomas, were included as a new category of neuroendocrine carcinomas in the 2010 World Health Organization classification. We recently experienced a rare case of a pancreatic carcinoma with both adenocarcinoma and neuroendocrine carcinoma components, a so-called mixed adenoneuroendocrine carcinoma.

**Patient concerns and diagnosis:**

A 66-year-old man was referred to our hospital with obstructive jaundice. Contrast-enhanced computed tomography images indicated a tumor located at the pancreatic head measuring 3.0 × 2.5 cm in diameter and invading the common bile duct. Cytological examination of the bile juice obtained by endoscopic retrograde cholangiopancreatography revealed adenocarcinoma cells. Pancreaticoduodenectomy was performed safely as radical resection.

**Interventions:**

Microscopically, the resected tumor consisted of tightly intermingled adenocarcinoma and neuroendocrine carcinoma components. On the immunohistochemical examination, p53 was ubiquitously positive in both components, whereas chromogranin A, synaptophysin and neuron-specific enolase, neuroendocrine markers, were limited to the neuroendocrine carcinoma component.

**Outcomes:**

Thus, such features of both adenocarcinoma and neuroendocrine carcinoma observed microscopically and immunohistochemically seemed to indicate a composite tumor.

**Lessons:**

The findings of this case suggest that adenocarcinoma and neuroendocrine carcinoma may be derived from a single cancer stem cell.

## 1 Introduction

In 2010, the World Health Organization (WHO) proposed a new classification of neuroendocrine neoplasms. In this classification, tumors with histological features of both adenocarcinoma and neuroendocrine carcinoma (NEC), each component exceeding 30%, are classified as mixed adenoneuroendocrine carcinomas (MANECs).^[1]^ MANECs have been reported in various organs including the colon,^[2,3]^ stomach,^[4,5]^ and biliary tracts.^[6–8]^ However, such tumors derived from the pancreas have rarely been reported.^[9]^

Recently, we experienced an extremely rare case of a MANEC derived from the pancreas. The clinical features and effective treatment of such tumors have not been well-described due to their rarity. Furthermore, because the histogenetic mechanisms underlying the development of mixed exocrine-endocrine tumors are not fully understood, we microscopically and histologically examined the components of this tumor using immunostaining, including chromogranin A, synaptophysin, neuron-specific enolase (NSE), and p53. In this report, we describe our immunohistochemical analysis in the hope that it may facilitate the development of new personalized therapeutic strategies.

## 2 Case report

### 2.1 Clinical case

**Figure 1:**
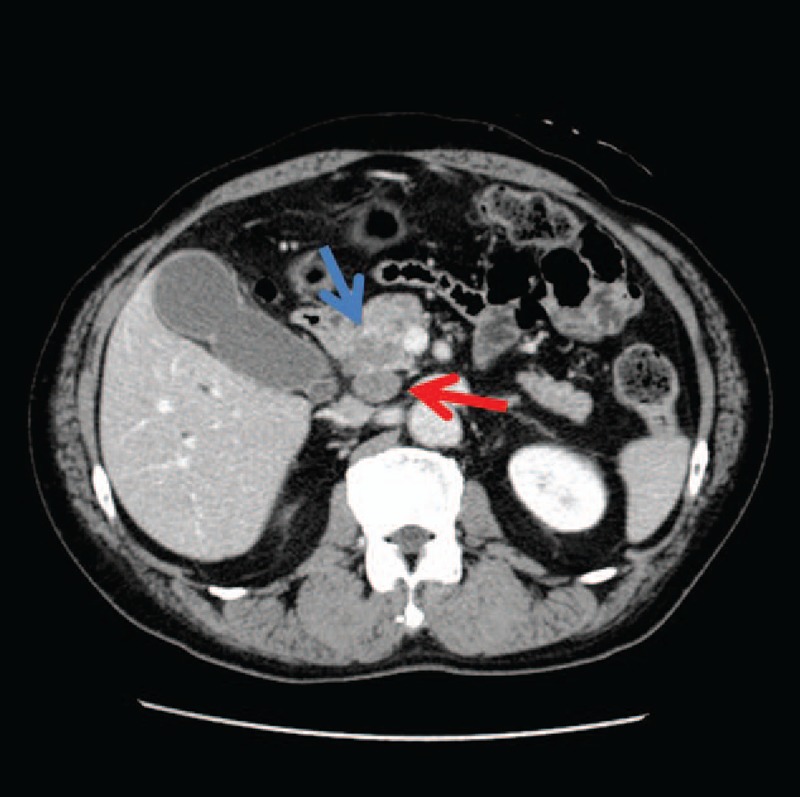
Contrast-enhanced computed tomography images showing a tumor in the pancreatic head measuring 3.0 × 2.5 cm in diameter and invading the common bile duct (indicated by a blue arrow). Lymphadenopathy was observed posterior to the pancreatic head (red arrow).

A 66-year-old man was referred to our hospital with obstructive jaundice. Laboratory examinations revealed elevated levels of serum total bilirubin of 8.35 mg/dL (normal, < 1.2 mg/dL) and serum carbohydrate antigen 19–9 of 95.6 U/mL (normal, < 37 U/mL). Contrast-enhanced computed tomography (CT) images demonstrated a tumor located in the pancreatic head measuring 3.0 × 2.5 cm in diameter and invading the common bile duct. The tumoral density was relatively low in the portal phase, and lymphadenopathy was observed posterior to the pancreatic head (Fig. 1); however, no distant metastasis was observed. Bile was aspirated using endoscopic retrograde cholangiopancreatography, and cytology revealed that it contained adenocarcinoma cells. The tumor was diagnosed as pancreatic carcinoma stage IIB (T3N1M0), according to the UICC classification of pancreatic cancer (7th edition).^[10]^ Pancreaticoduodenectomy with Japanese D2 lymph node (LN) dissection was performed safely with informed consent, and the patient was discharged on 32nd post-operative day without any adverse events. The patient administered 2 courses of Tegafur gimercil oteracil (S-1) monotherapy (80 mg/m^2^/day for 4 weeks and then stopped for 2 weeks of a 6-week cycle) as adjuvant chemotherapy. He died 12 months postoperatively due to multiple liver metastases unaffected by 2 courses of chemotherapy comprising S-1 and gemcitabine (S-1 65 mg/m^2^/day on days 1 through 14 plus gemcitabine 1000 mg/m^2^ on days 1 and 8 of a 21-day cycle).

### 2.2 Microscopic and immunohistochemical findings

The resected specimen, including the pancreatic head tumor and regional LNs, was fixed in 10% neutral-buffered formalin and embedded in paraffin. From these blocks, 4-μm-thick sections were examined microscopically with hematoxylin and eosin (H&E) staining and immunohistochemically using the antibodies carcinoembryonic antigen (CEA) (monoclonal antibody, Ventana Medical Systems, Tucson, AZ), chromoglanin A (primary antibody, Ventana Medical Systems), synaptophysin (monoclonal antibody, Ventana Medical Systems), NSE (monoclonal antibody, Ventana Medical Systems), and p53 (primary antibody, Ventana Medical Systems). Immunostaining was performed fully automatically using a Ventana Bench Mark GX (Roche, Inc., Germany).

**Figure 2:**
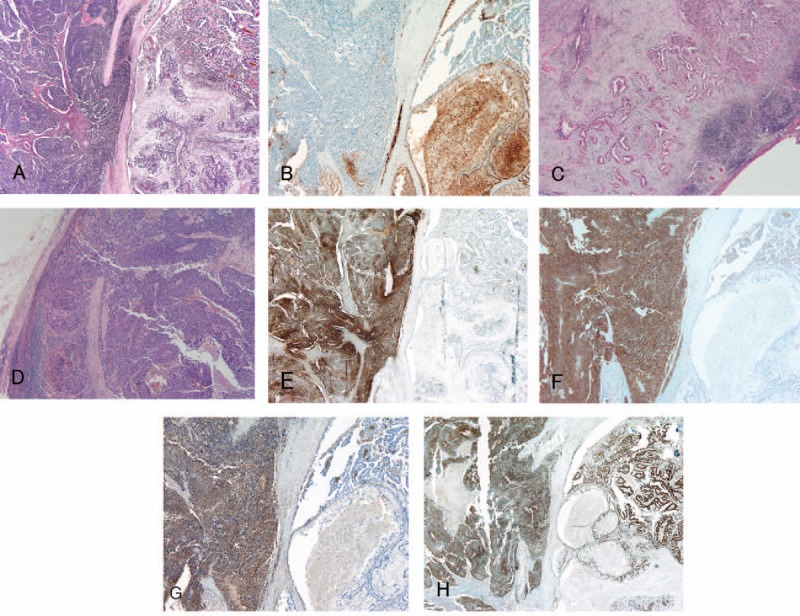
Microscopic and immunohistochemical appearance of the tumor. (A) Primary tumor (hematoxylin and eosin staining, original magnification ×20). The primary lesion was composed of a well-differentiated adenocarcinoma component and a poorly differentiated NEC component, each tightly intermingled. (B) Immunohistochemical staining of the primary lesion for CEA (original magnification ×20). CEA was limited to the well-differentiated adenocarcinoma component of the primary lesion. (C) LN no. 12 exclusively contained the well-differentiated adenocarcinoma component (original magnification ×20). (D) LN no. 13 exclusively contained the poorly differentiated NEC component (original magnification ×20). (E) Immunohistochemical staining of the primary lesion for chromogranin A (original magnification ×20). Chromogranin was limited to the poorly differentiated NEC component of the primary lesion. (F) Immunohistochemical staining of the primary lesion for synaptophysin (original magnification ×20). Synaptophysin was limited to the poorly differentiated NEC component of the primary lesion. (G) Immunohistochemical staining of the primary lesion for NSE (original magnification ×20). NSE was limited to the poorly differentiated NEC component of the primary lesion. (H) Immunohistochemical staining of the primary lesion for p53 (original magnification ×20). p53 was ubiquitously expressed in both the well-differentiated adenocarcinoma and poorly differentiated NEC components of the primary lesion. The well-differentiated adenocarcinoma component can be seen on the right sides and the poorly differentiated NEC component can be seen on the left (E, F, G, and H). CEA = carcinoembryonic antigen, LN = lymph node, NEC = neuroendocrine carcinoma, NSE = neuron-specific enolase.

Microscopically, the tumor had a well-differentiated adenocarcinoma component as well as a poorly differentiated adenocarcinoma component, indicating NEC (Fig. 2A). The well-differentiated adenocarcinoma component was predominant, and each component was tightly intermingled. Well-differentiated carcinoma and poorly differentiated NEC components occupied approximately 60% and 40% of the tumor, respectively. Well-differentiated adenocarcinoma cells were seen to proliferate in a papillary and tubular fashion indicating ductal adenocarcinoma, whereas the poorly differentiated NEC demonstrated a trabecular growth pattern with sheets of small carcinoma cells (small cell type). In addition, CEA was expressed in well-differentiated adenocarcinoma cells (Fig. 2B). Both components were accompanied with lymphatic, venous, and perineural invasion, particularly in infiltrative regions. In both components, mitotic cells were frequent (21 mitoses per 10 high-power fields) and a Ki-67 labeling index (LI) was approximately 40% (data not shown). A proportion of dissected regional LNs revealed invasion by cancer cells; LNs no. 8p, 12b, and 17a (according to the Japanese general rules for the study of pancreatic cancer)^[11]^ were invaded solely by well-differentiated carcinoma cells (Fig. 2C), whereas LN no. 13 was invaded solely by poorly differentiated NEC cells (Fig. 2D).

Immunohistochemically, the neuroendocrine markers chromogranin A, synaptophysin, and NSE were limited to the NEC component of the primary tumor (Fig. 2E–G). In contrast, p53 was ubiquitously expressed in both components of the primary tumor (Fig. 2H), and LN no. 13, containing poorly differentiated NEC, was immunohistochemically positive for chromogranin A, synaptophysin, and NSE. LNs no. 8p, 12b, and 17a, containing well-differentiated adenocarcinomas, were negative for such markers. Furthermore, each LN containing well-differentiated adenocarcinomas or poorly differentiated NEC was immunohistochemically positive for p53, as in the primary lesion (data not shown).

## 3 Discussion

The tumor that is the subject of this study contained well-differentiated adenocarcinoma and poorly differentiated NEC components, accompanied by a high mitotic rate and a high Ki-67 LI in both components, consistent with the diagnostic criteria for MANEC.^[1]^ Thus, we report here on a rare case of a MANEC. In general, tumors with multiple phenotypic features, regardless of their oncogenesis, are considered to have highly malignant biological behavior.^[12]^ Indeed, consistent with these reports, our patient died due to multiple liver metastases 12 months after pancreaticoduodenectomy. To date, the clinical features and effective treatments of such tumors have not been well-described, and the histogenetic mechanisms underlying the development of mixed exocrine-endocrine tumors have not been fully elucidated. In the present case, we examined each component microscopically and immunohistologically using chromogranin A, synaptophysin, NSE and p53.

Microscopically, each component of the tumor discussed here was tightly intermingled, similar to previously reported cases derived from various organs, including the biliary and gastrointestinal tracts.^[2–8]^ Such features indicate that different components may derive from a single cancer stem cell. MANECs can be divided into 3 subtypes such as composite, collision, and amphicrine tumors.^[13]^ Immunohistochemically, p53 was ubiquitously positive in both components, whereas the expression of chromogranin A, synaptophysin and NSE, neuroendocrine markers, was limited to the NEC component, indicating a composite tumor on the basis of microscopic findings. Furthermore, these results suggest that p53 gene mutation was common to both components, and an additional gene alteration resulted in the phenotypic expression of chromogranin A, synaptophysin, and NSE. In light of the results of immunohistochemical analysis, both components in this case may derive from a single cancer stem cell and the NEC component may be differentiated from the well-differentiated adenocarcinoma component.

Interestingly, some LNs contained just well-differentiated adenocarcinoma components, whereas others contained just poorly differentiated NEC components. In addition, affected LNs demonstrated similar immunohistochemical staining patterns to the primary lesion for chromogranin A, synaptophysin, NSE, and p53. These microscopic and immunohistochemical features may be attributable to individual and similar malignant behaviors of each component, including lymphatic, venous, and perineural invasion, particularly in the infiltrative regions of the primary lesion. A pathological autopsy was not performed in this case as informed consent was not obtained, and hence the histological features of the liver metastases were not revealed. However, liver metastases may have contained both well-differentiated carcinomas and poorly differentiated NEC because lymphatic, venous, and perineural invasion were observed in both components of the primary lesion and the presence of each component in affected LNs.

Regarding the treatment of tumors characterized by the typical microscopic features of MANEC and highly malignant biological behavior, as in this case, a surgical approach alone appears to be insufficient. S-1,^[14]^ gemcitabine,^[15]^ irinotecan,^[16]^ oxaliplatin,^[16]^ cisplatin,^[17]^ nab-paclitaxel,^[18]^ and erlotinib^[19]^ are currently available for metastatic pancreatic cancer chemotherapy, either alone or in combination as adjuvant therapy. Of these, we selected S-1 for adjuvant chemotherapy,^[20]^ and S-1 and gemcitabine for chemotherapy of the liver metastases, according to the clinical diagnosis of pancreatic cancer. Unfortunately, the patient died 12 months postoperatively because of multiple liver metastases that were unaffected by chemotherapy. In hindsight, because of the microscopic features and histogenesis of the present tumor, a different combination of anticancer drugs, such as irinotecan and cisplatin, effective for the treatment of small cell lung cancer, may have been more effective. Microscopically, NEC resembles small cell lung cancer and such a combination of anticancer drugs has previously been reported to be effective in treating NEC of the pancreas.^[21,22]^ Furthermore, as the histogenesis of both components may have derived from a common origin, an effective combination of anticancer drugs for NEC may be feasible for the treatment of the well-differentiated adenocarcinoma component.

## 4 Conclusion

We report a rare case of pancreatic MANEC with a poor prognosis. Immunohistochemical analyses support the hypothesis that the NEC component may have given rise to the well-differentiated adenocarcinoma component, with both components potentially derived from a single cancer stem cell. Further examination, and the accumulation of reports of similar malignant tumors, may facilitate the development of multimodal treatments, including neoadjuvant chemoradiation therapy and/or tailor-made adjuvant chemotherapy for each component combined with radical resection.
